# Robust TLR4-induced gene expression patterns are not an accurate indicator of human immunity

**DOI:** 10.1186/1479-5876-8-6

**Published:** 2010-01-27

**Authors:** Kelly L Brown, Reza Falsafi, Winnie Kum, Pamela Hamill, Jennifer L Gardy, Donald J Davidson, Stuart Turvey, Brett B Finlay, David P Speert, Robert EW Hancock

**Affiliations:** 1 Centre for Microbial Diseases and Immunity Research, Department of Microbiology and Immunology, University of British Columbia, Vancouver, British Columbia, V6T 1Z3, Canada; 2 Child and Family Research Institute, University of British Columbia, 950 West 28th Avenue, Vancouver, British Columbia, V5Z 4H4, Canada; 3 Current address: MRC/University of Edinburgh Centre for Inflammation Research, Queen's Medical Research Institute W2 03, 47 Little France Crescent, Edinburgh EH16 4T, UK; 4 Current address: Department of Rheumatology and Inflammation Research, University of Gothenburg, Guldhedsgatan 10, S-413 46 Gothenburg, Sweden

## Abstract

**Background:**

Activation of Toll-like receptors (TLRs) is widely accepted as an essential event for defence against infection. Many TLRs utilize a common signalling pathway that relies on activation of the kinase IRAK4 and the transcription factor NFκB for the rapid expression of immunity genes.

**Methods:**

21 K DNA microarray technology was used to evaluate LPS-induced (TLR4) gene responses in blood monocytes from a child with an IRAK4-deficiency. *In vitro *responsiveness to LPS was confirmed by real-time PCR and ELISA and compared to the clinical predisposition of the child and IRAK4-deficient mice to Gram negative infection.

**Results:**

We demonstrated that the vast majority of LPS-responsive genes in IRAK4-deficient monocytes were greatly suppressed, an observation that is consistent with the described role for IRAK4 as an essential component of TLR4 signalling. The severely impaired response to LPS, however, is inconsistent with a remarkably low incidence of Gram negative infections observed in this child and other children with IRAK4-deficiency. This unpredicted clinical phenotype was validated by demonstrating that IRAK4-deficient mice had a similar resistance to infection with Gram negative *S. typhimurium *as wildtype mice. A number of immunity genes, such as chemokines, were expressed at normal levels in human IRAK4-deficient monocytes, indicating that particular IRAK4-independent elements within the repertoire of TLR4-induced responses are expressed.

**Conclusions:**

Sufficient defence to Gram negative immunity does not require IRAK4 or a robust, 'classic' inflammatory and immune response.

## Background

Toll-like receptor-4 (TLR4) is a prominent member of the TLR family of host receptors that recognize microbial components in the intra- and extra-cellular environment [1,2]. Lipopolysaccharide (LPS, endotoxin) is a major component of the cell wall of Gram negative bacteria, a potent TLR4 agonist and the driving force behind sepsis. TLR4 engagement by LPS results in the activation of the transcription factor NFκB via signal transduction cascades that are propagated either through, or independent of, the adaptor molecule MyD88 [2-4]. Organisms amenable to genetic manipulation have been used to evaluate the importance of TLR4 and various downstream signalling molecules for the (LPS-)induced expression of immunity genes (most commonly cytokines including chemokines) and defence against various pathogens [5,6], e.g., MyD88-knockout mice fail to elevate serum cytokines when administered a high dose of LPS and are susceptible to infection by *S. aureus *[7], *P. aeruginosa *[8], *M. tuberculosis *[9], *M. avium *[10] and *L. monocytogenes *[11]. Such studies have established that the MyD88-dependent pathway, via sequential activation of IRAK4, IRAK1, TRAF6, IKK and NFκB, drives a cellular response to TLR agonists that is responsible for the robust expression of early-response, NFκB-regulated immunity genes. It has been often assumed that this robust transcriptional response, involving the substantial induction of a large number of genes (>1000) is essential for normal immunological function(s) and, in turn, host defences. Moreover, the reduced expression, either *in vitro *or *in vivo*, of just a subset of TLR-responsive genes, such as the classic pro-inflammatory cytokines (TNF-α, IL-6), chemokines (IL-8, Gro-α/CXCL1) and antibacterials (antimicrobial peptides, reactive oxygen/nitrogen species) is often accepted as sufficient evidence to predict a defect in host defence against infectious agents.

Since 2003, eighteen individuals, primarily children, have been identified with a mutation in the gene that encodes IL-1R-associated kinase 4 (IRAK4) that lies immediately downstream of MyD88 in the TLR signalling cascade (reviewed in [12]). We previously reported an IRAK4-deficiency in a 4-year old child due to a homozygous single base substitution (C887T) in exon 8 of the IRAK4 gene that introduced a premature stop codon (Q293X) [13,14]; an identical mutation occurs in the majority of cases of IRAK4 immunodeficiency. PBMC from children with IRAK4-deficiency consistently show impaired *in vitro *responsiveness, as measured by the production of pro-inflammatory cytokines, to selected TLR agonists [12-14]. Given the pivotal position of IRAK4 in the TLR pathway and the failed responsiveness of IRAK4-deficient (human and mouse) cells to both Gram positive (LTA, PGN) and Gram negative (LPS) TLR agonists, it seemed logical that a defect in IRAK4 would render patients susceptible to a broad range of both Gram positive and Gram negative bacterial pathogens. In contrast, children with an IRAK4-deficiency have a remarkably mild immunodeficiency with enhanced susceptibility to infection by only a narrow range of Gram positive bacteria, particularly *S. aureus *and *S. pneumoniae *[12-18]. IRAK4 knockout mice are also more susceptible to infections by (Gram positive) *S. aureus *than wild-type mice [19]. We were intrigued by the relatively small number of documented cases of Gram negative infections in IRAK4-deficient children [20,21] in light of a consistently poor cellular responses to LPS. It should be noted that these children are prescribed a regimen of antibiotics that are effective only against Gram positive bacteria, thus prophylaxis can not explain their apparent immunity to Gram negative infections. IRAK4 knockout mice and IRAK4-kinase-dead knock-in mice also fail, *in vitro *and *in vivo*, to produce inflammatory cytokines in response to LPS and are protected against LPS-induced sepsis *in vivo *[19,22-24]. There is, however, a resounding lack of experimental evidence that IRAK4-deficiency (in humans or mice) actually compromises host defences to Gram negative infection.

Herein we provide the first evidence that IRAK4 knockout mice are not overwhelmed by challenge with the Gram negative bacterium *S. typhimurium*. These results substantiated our hypothesis that the underwhelming number of documented Gram negative bacterial infections in IRAK-deficient children is likely attributable to sufficient host defence. The widely accepted notion that the absence of IRAK4 should compromise immunity to Gram negative pathogens was based primarily upon results demonstrating the compromised expression of a small number of classic LPS-responsive, pro-inflammatory markers (primarily cytokines) as well as the assumption that high expression of these genes in particular was required for immunity. Here we evaluated the impact of IRAK4-deficiency on a global scale using 21 K microarray technology to analyze total gene expression in LPS-activated monocytes from an IRAK4-deficient patient. In accordance with previous reports, and counter-intuitive to a resistance to Gram negative infection, there was a profound failure of IRAK4-deficient monocytes to produce, in response to LPS, substantial levels of classical immunity genes (cytokines, chemokines, NFκB subunits). Upon closer examination, however, it was discovered that the expression of these genes, while low, was seldom abolished. Moreover, approximately 20% of LPS-responsive genes, including certain chemokines, transcription factors and regulators of translation, were expressed at similar levels in control and IRAK4-deficient cells. We conclude that IRAK4 is indeed essential for LPS-induced signal transduction via the MyD88-dependent pathway that is responsible for rapid and vigorous transcription of classical NFκB-regulated immunity genes, but that neither IRAK4 nor a robust transcriptional response (typical of cells exposed to LPS) are required for immunity to Gram negative infections.

## Methods

### PBMC isolation and stimulation

PBMC from healthy volunteers and the patient were prepared as previously described [25], in accordance with UBC Clinical Research Ethics Board protocol C04-0193. Due to the rarity of this syndrome only a single patient was available to us; however previous studies have demonstrated similar phenotypic responses for most IRAK4 deficient patients including this one [13-18]. PBMC (2-3 × 10^7 ^cells at 5 × 10^6 ^cells/ml) were stimulated for 4 hr at 37°C 5% CO_2 _with 100 ng/ml *Escherichia coli *0111:B4 LPS (Invivogen). Based on previous studies, LPS-induced gene transcription in monocytes peaked (with respect to the number of genes and the magnitude of gene expression) after 4 hr of stimulation, at which point a substantial level of inflammatory cytokines could also be detected in the tissue culture supernatant [25]. We previously reported comparable cytokine responses in PBMC isolated from adults and children following *in vitro *stimulation with TLR agonists [26]; thus for ethical reasons we used adult PBMC as controls in these experiments. All reagents were tested for the absence of endotoxin and reconstituted in endotoxin-free water.

### Detection of cytokines and chemokines

Following culture of PBMC, the tissue culture supernatants were centrifuged and stored at -80°C and/or measured for cytokines using a cytokine 5-Plex kit (Biosource International Inc) and Luminex 100™ StarStation software (Applied Cytometry Systems) as described [25]. CCL22 (MDC) secretion in tissue culture supernatants was detected with a capture ELISA (R&D Systems).

### Positive selection of CD14^+ ^monocytes and DNA microarrays

Following culture of PBMC, monocytes were positively selected using anti-CD14 conjugated magnetic beads (M450; Dynal; Invitrogen) as described [27,28]. RNA was isolated from monocytes with RNeasy Mini kit and analyzed using an Agilent 2100 Bioanalyzer (Agilent Technologies) as described [25]. Equal quantities of RNA from each of five healthy individuals were pooled per experimental condition to generate a reference sample of the average (n = 5) expression of genes in control monocytes. The profile of LPS-responsive gene expression in the control pool was consistent with what has been observed by us in monocytes from individual subjects [29]. Microarray analyses were performed on 21,000 gene arrays as previously described [29], analyzed by ArrayPipe software, version 1.6 [30], and the data deposited into ArrayExpress under accession number E-FPMI-7. Differentially expressed genes were overlayed on known signal transduction pathways using Cytoscape, an open-source bioinformatics visualization software [31]. The Gene Ontology Tree Machine software [32] was used to identify gene ontology (GO) categories and biological processes with a significantly enriched (ratio >1.0 and p < 0.01) number of differentially expressed genes (indicative of dysregulation) in the patient's monocytes.

### Quantitative real-time PCR (qPCR)

Differential gene expression was validated using the SuperScript™ III Platinum^® ^Two-Step qRT-PCR Kit with SYBR^® ^Green (Invitrogen) [25], using 10 ng of total RNA as the starting material. Reported fold changes in gene expression were normalized to GAPDH in each sample and were relative to the expression of the gene in resting control monocytes. The result of qPCR analyses of more than twenty, TLR-inducible, NFκB-regulated genes was in agreement with the microarray gene expression data 84% of the time; a correlation indicative of a reliable microarray data set with a relatively low incidence of false results.

### Bacterial culture and infection of mice

IRAK4^-/- ^mice on the C57BL/6 background [19] were obtained from the Canadian Network for Vaccines and Immunotherapeutics of Cancer and Chronic Viral Diseases (CanVac). Sex- and age-matched control C57BL/6 mice were purchased from Jackson Laboratories (Bar Harbor, Maine). Animals were used at 8-10 weeks of age. Studies were performed under pathogen-free conditions according to the standard animal care guidelines and protocols of the UBC Animal Care Committee and Canadian Council on Use of Laboratory Animals. *Salmonella *serovar Typhimurium wild-type strain SL1344 [33] was grown with overnight shaking (220 rpm) in 3 ml Luria-Bertani (LB) broth with 50 μg/ml streptomycin at 37°C for 18 hr. Groups of eight mice were infected by oral gavage with approximately 1 × 10^7 ^CFU in 100 μl of sterile PBS. Infected mice were monitored twice daily. Mice that showed extreme distress or became moribund were euthanized and survival of the animals was recorded.

## Results

### IRAK4-deficient mice survive infection by the Gram negative pathogen Salmonella typhimurium

**Figure 1:**
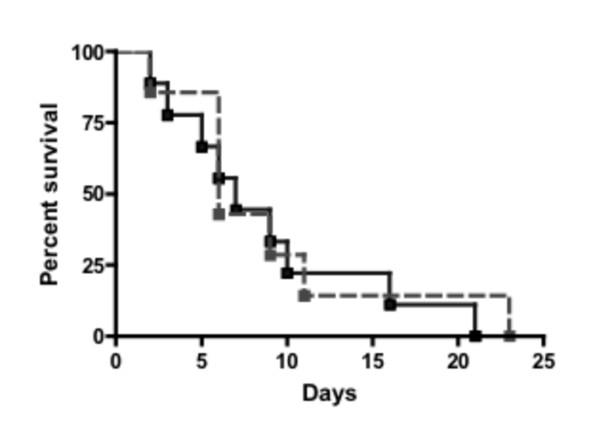
**IRAK4-deficient mice maintain defence against Gram negative bacteria**. IRAK4 ^-/- ^(dotted line) and age- and sex-matched C57BL wild type (solid line) mice were infected with *S. typhimurium *SL1344 and monitored for survival (y-axis) over time (x-axis). The result is representative of two independent experiments where lack of statistical significance (p = 0.69 and p = 0.13) for differential survival between two groups of eight infected mice was determined by the chi-square test.

To validate the unusually low occurrence of Gram-negative infections in IRAK4-deficient children, IRAK4-deficient mice were challenged with the Gram negative bacterium, *S. typhimurium*. Results shown in Figure 1 demonstrated no significant difference in survival rates between IRAK4-deficient mice and wild type mice following challenge with *S. typhimurium*. Thus, the absence of IRAK4 does not render animals defenceless against a Gram negative bacterial challenge. These data together with that of Suzuki *et al *demonstrated that IRAK4 knockout mice are highly susceptible to Gram positive [19] but not necessarily Gram negative bacterial infections (Figure 1), an infection profile that emulates that of children with an IRAK4 deficiency. Thus these data strengthen our hypothesis that immunity, as opposed to prophylaxis or a lack of exposure to Gram negative bacteria, is responsible for the underwhelming number of documented Gram negative infections in children with IRAK4-deficiency.

### Gene expression was generally severely compromised in IRAK4-deficient monocytes stimulated with LPS

The long-standing assumption that any deficiency in the major TLR to NFκB pathway (such as an IRAK4-deficiency) would compromise immunity to Gram negative bacteria [34-36] was based on a substantial amount of published data demonstrating compromised expression *in vitro *of a limited set of LPS-induced, early-response, NFκB-regulated, pro-inflammatory markers in cells deficient for various components of the TLR to NFκB pathway (including IRAK4). A functional genomics approach was utilized here to view the broader impact of an IRAK4 deficiency on LPS-induced gene expression. Blood monocytes were obtained from an IRAK4-deficient child and subsequently stimulated *in vitro *in two independent experiments, two months apart. With human 21 K oligo-based DNA microarray technology, the expression of genes in IRAK4-deficient monocytes from each sampling was evaluated, in triplicate, relative to gene expression in monocytes from five healthy individuals. Previous studies that employed a similar methodology [28,29] served as a reference to confirm normal LPS-responsive gene expression in the healthy, control pool of monocytes.

**Figure 2:**
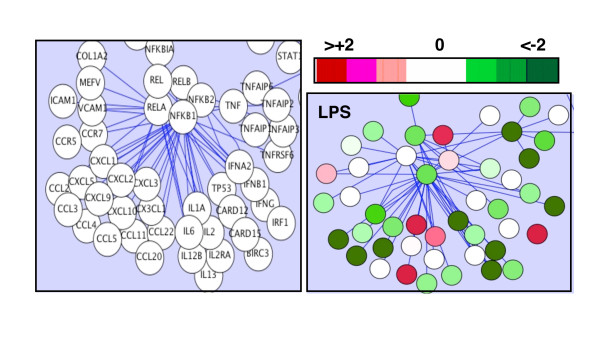
**Differentially expressed genes in LPS-stimulated IRAK4-deficient monocytes downstream of NFκB**. Using the open-source bioinformatics visualization software Cytoscape [31] gene expression in LPS-stimulated, IRAK4-deficient monocytes was overlayed on the TLR4 signal transduction pathway downstream of NFκB. Gene names are given in the left panel and gene expression in the right panel. The relative expression of the gene in IRAK4-deficient monocytes is depicted by the colour of the nodes on the protein map; green nodes indicate lower expression and red nodes indicate higher expression in IRAK4-deficient monocytes relative to that in a control pool of monocytes.

The expression of more than 500 genes in IRAK4-deficient cells stimulated with LPS was significantly different compared to that in control cells (567 genes, differential fold change ± 1.5, Student's t-test p-value ≤ 0.05, results deposited in ArrayExpress, accession number E-FPMI-7). An overlay of the gene expression data on a protein map of the TLR-to-NFκB signal transduction pathway illustrated that the majority of differentially expressed genes associated with this pathway were i) suppressed relative to gene expression in control monocytes and ii) tended to congregate downstream of the IκB-NFκB complex (Figure 2). Gene ontology (GO) analysis [32] of the differentially expressed genes predicted that 56 GO categories were perturbed in IRAK4-deficient patients; these constituted 5 major biological processes (p < 0.01) namely 'immune response', 'response to biotic stimulus', 'response to stress', 'cell adhesion' and 'negative regulation of cellular processes' (data not shown).

Thus, the microarray analysis of monocytes from an IRAK4-deficient child revealed a substantial disturbance in LPS-induced gene expression, in particular in genes regulated by the TLR-to-NFκB pathway and associated with biological processes related to immunity and cellular responsiveness to stimuli. This global analysis of gene expression in IRAK4-deficient monocytes was in accordance with previously published results that analyzed small subsets of inflammatory genes/proteins and confirmed the previously ascribed role of IRAK4 in mediating signalling from TLR4 to genes downstream of NFκB.

### Expression of NFκB-regulated, pro-inflammatory genes was compromised to varying degrees in IRAK4-deficient monocytes

Cytokines, chemokines and certain antimicrobial peptides are TLR-sensitive, early response genes of the innate immune system that are commonly used to predict host defence and infectious disease risk. A subset of these TLR-to-NFκB-regulated immunity genes revealed in the microarray analyses were selected for further investigation. Genes were selected if they fit one or more of the following criteria (i) the gene was regulated by cellular activation with LPS (ii) the gene was a classic pro-inflammatory mediator, for e.g., a cytokine (TNF-α, IL-6), chemokine (IL-8, Gro-α/CXCL1) or antibacterial (DEFB1) (iii) in previously published work, alteration of the expression of the gene or protein product, either *in vitro *or *in vivo*, was predicted to influence host defence, or (iv) the gene served as an internal control (for e.g., induction of NFκB subunits). Real-time quantitative PCR (qPCR) and ELISA were used to confirm the microarray results for relative gene expression between control and IRAK4-deficient monocytes, and to establish the absolute expression of mRNA and protein prior to and following stimulation with LPS.

**Figure 3:**
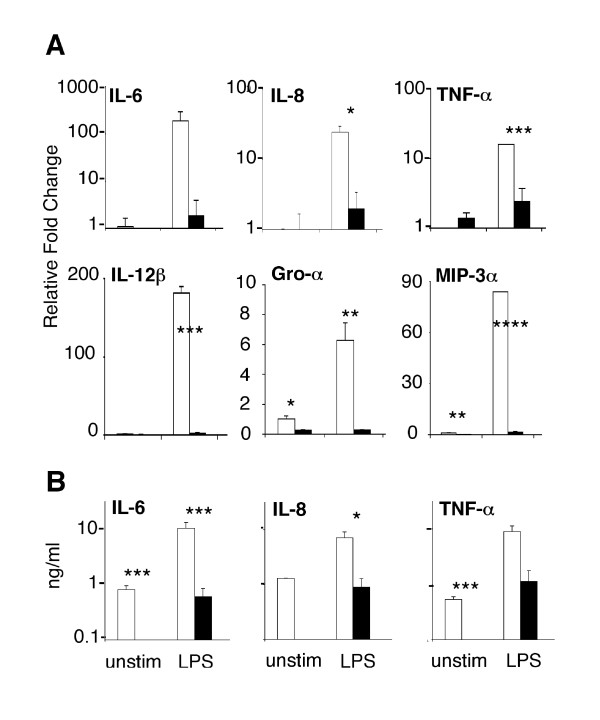
**Expression of inflammatory cytokines and chemokines in IRAK4-deficient monocytes**. A Expression of IL-6, IL-8, IL-12β, TNF-α, Gro-α/CXCL1 and MIP-3α/CCL20 in the healthy control pooled monocytes (white bars) and in both biological replicates of the patient's monocytes (black bars) were evaluated, in duplicate, by qPCR following a 4 hr incubation in the absence or presence of LPS (X-axis). **B **IL-6, IL-8 and TNF-α were also measured in the tissue culture supernatant of stimulated PBMC by multiplex cytokine bead immunoassays. The Y-axis represents fold change (log scale) in gene expression relative to unstimulated control monocytes (qPCR) or pg/ml of protein determined by the cytokine assay. Differences in expression or protein secretion were determined to be statistically significant by Student's two tailed T-test with a value of p < 0.15 (*), p < 0.1 (**), p < 0.05 (***) and p < 0.003 (****).

The expression of classic pro-inflammatory cytokines and chemokines including *TNF-α*, *IL-6*, *IL-12β*, *IL-8*, *Gro-α/CXCL1*, *MCP-2/CCL8 *and *MIP-3α/CCL20 *was compromised in LPS-stimulated IRAK4-deficient monocytes compared to LPS-stimulated control monocytes (Figure 3 and Table 1). While the expression of these genes was unanimously compromised, the degree of suppression varied considerably, from 5- to 100- fold reduction in expression (Figure 3A, Table 1). The relative level of protein expressed by PBMC was also heavily compromised (ELISA shown for TNF-α, IL-8, IL-6 in Figure 3B). These data were consistent with previous reports of low levels of cytokines detected in the TCS of LPS-stimulated PBMC from this patient and other individuals with mutations in the IRAK4 gene [12-18] as well as diminished, but not absent levels of TNF-α and IL-6 in the serum of IRAK4-kinase dead (KD) knockin mice following *in vivo *administration of LPS [37]. These data (Figure 2, Figure 3, Table 1) illustrated that gene transcription and protein secretion of key pro-inflammatory mediators, while highly variable, was *not *completely abolished in IRAK4-deficient monocytes exposed to LPS.

**Table 1:** Gene expression by qPCR in resting or LPS-activated IRAK4-deficient monocytes relative to gene expression in unstimulated control cells

Gene Name	Monocytes	Resting		LPS	
DEFB1	Control	^a^1.1 ± 0.6		12.6 ± .0	
			**up**		**=**
	*IRAK4 Q293X*	*1.9 ± 0.9*		*9.6 ± 0.9*	

Fos	Control	1.5 ± 0.1		0.3 ± 0.0	
			**=**		**=**
	*IRAK4 Q293X*	*1.3 ± 0.0*		*0.3 ± 0.1*	

Gro-α (CXCL1)	Control	1.0 ± 0.2		6.3 ± 1.2	
			dn		dn
	*IRAK4 Q293X*	*0.3 ± 0.0*		*0.3 ± 0.0*	

IL-6	Control	1.1 ± 0.6		185.4 ± 110.0	
			dn		dn
	*IRAK4 Q293X*	*0.4 ± 0.5*		*1.8 ± 2.1*	

IL-8	Control	1.0 ± 0.0		24.1 ± 5.5	
			**=**		dn
	*IRAK4 Q293X*	*0.9 ± 0.8*		*2.0 ± 1.4*	

IL-12β	Control	1.0 ± 0.4		181.7 ± 8.0	
			dn		dn
	*IRAK4 Q293X*	*0.2 ± 0.1*		*1.9 ± 0.8*	

IL-13RA1	Control	1.0 ± 0.1		0.3 ± 0.0	
			**=**		**up**
	*IRAK4 Q293X*	*1.1 ± 0.3*		*1.9 ± 0.1*	

IFN-γ	Control	1.0 ± 0.1		11.0 ± 0.3	
			dn		dn
	*IRAK4 Q293X*	*0.4 ± 0.1*		*0.8 ± 0.3*	

IRF1	Control	1.0 ± 0.1		6.7 ± 0.1	
			dn		dn
	*IRAK4 Q293X*	*0.6 ± 0.1*		*3.1 ± 0.2*	

IκBζ	Control	1.1 ± 0.5		1.3 ± 0.3	
			dn		dn
	*IRAK4 Q293X*	*0.4 ± 0.2*		*0.5 ± 0.1*	

Jun	Control	1.0 ± 0.0		0.9 ± 0.2	
			**=**		**up**
	*IRAK4 Q293X*	*0.9 ± 0.3*		*1.7 ± 0.4*	

MCP-2 (CCL8)	Control	1.0 ± 0.0		9.4 ± 0.0	
			dn		dn
	*IRAK4 Q293X*	*0.1 ± 0.0*		*1.8 ± 0.2*	

MDC (CCL22)	Control	1.1 ± 0.5		4.1 ± 1.2	
			dn		**up**
	*IRAK4 Q293X*	*0.6 ± 0.2*		*6.4 ± 1.8*	

MIP-3α (CCL20)	Control	1.0 ± 0.2		83.9 ± 0.0	
			dn		dn
	*IRAK4 Q293X*	*0.1 ± 0.0*		*1.4 ± 0.5*	

NFκB1 (p105/p50)	Control	1.0 ± 0.1		6.8 ± 0.1	
			dn		dn
	*IRAK4 Q293X*	*0.3 ± 0.1*		*2.3 ± 0.7*	

NFκB2 (p100/p52)	Control	1.0 ± 0.0		4.5 ± 0.7	
			dn		dn
	*IRAK4 Q293X*	*0.7 ± 0.0*		*1.9 ± 0.3*	

NFκB cRel	Control	1.0 ± 0.1		3.8 ± 0.1	
			dn		dn
	*IRAK4 Q293X*	*0.7 ± 0.0*		*1.0 ± 0.0*	

NFκB RelA (p65)	Control	1.0 ± 0.0		3.3 ± 0.2	
			**=**		dn
	*IRAK4 Q293X*	*1.2 ± 0.0*		*2.3 ± 0.4*	

NFκB RelB	Control	1.0 ± 0.2		1.9 ± 0.4	
			**=**		**=**
	*IRAK4 Q293X*	*0.8 ± 0.0*		*1.7 ± 0.3*	

SOCS1	Control	1.2 ± 0.8		21.2 ± 4.7	
			dn		dn
	*IRAK4 Q293X*	*0.1 ± 0.1*		*1.6 ± 0.5*	

TNF-α	Control	1.0 ± 0.1		11.3 ± 4.0	
			**=**		dn
	*IRAK4 Q293X*	*0.9 ± 0.6*		*2.4 ± 0.7*	

ZFP36L2	Control	1.0 ± 0.1		0.8 ± 0.0	
			**up**		**up**
	*IRAK4 Q293X*	*2.1 ± 0.5*		*2.1 ± 0.2*	

^a ^fold changes normalized to endogenous GAPDH in patient or control cells then expressed relative to that in the pool of unstimulated control monocytes (fold change = 1.0) ± the standard deviation of 2 biological and 2 technical replicates.

### Identification of LPS-responsive, IRAK4-independent immunity genes

While LPS-induced expression of the majority of NFκB-regulated genes was lowered in IRAK4-deficient monocytes, the TLR-to-NFκB pathway map (shown in Figure 2) revealed a subset of genes that were expressed at similar (white nodes) or higher (red nodes) levels in IRAK4-deficient and control cells. It was discovered that approximately 20% of LPS-responsive genes were expressed in IRAK4-deficient monocytes in a similar manner to that observed in control cells. A subset of LPS-responsive genes associated with host defence that were induced or suppressed to a similar extent (<1.2 fold difference) in control and IRAK4-deficient monocytes are listed in Table S1, Additional file 1 (microarray data). Genes of this nature encoded a diverse range of proteins involved in immunity including, but not restricted to, chemokines (*MDC/CCL22*), antibacterial agents (*DEFB1*), transcription factors (*FOS, JUN, RELB*), cytokine receptors (*IL13RA1*) and mRNA-destabilizing agents (*ZFP36L2*). The uncompromised expression of these genes in IRAK4-deficient cells was confirmed by qPCR (Table 1).

**Figure 4:**
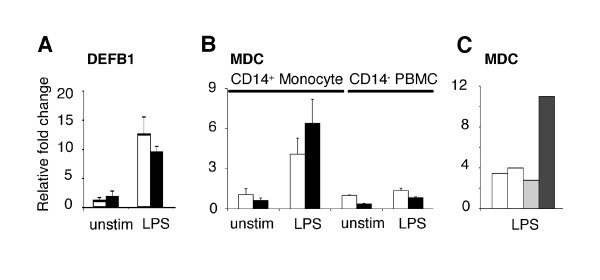
**Strong expression of particular chemokines in IRAK4-deficient monocytes**. The relative expression (Y-axis) of (**A**) DEFB1 and (**B**) MDC/CCL22 in IRAK4-deficient monocytes (black bars), controls (white bars) and for B) CD14^- ^PBMC. Expression was measured in two independent experiments by real-time PCR following 4 hr incubation in the absence or presence of LPS (X-axis). **C **MDC/CCL22 in the tissue culture supernatant was measured by ELISA in IRAK4-deficient (black bars), adult (white bars) or age-matched (grey bars) PBMC. The result is representative of 2 independent experiments and the y-axis represents the relative fold change in protein after stimulation for 24 hr with LPS.

The relatively normal expression of the chemoattractant *MDC/CCL22 *and the chemotactic host defence (antimicrobial) peptide defensin-β-1 (*DEFB1*) in IRAK4-deficient monocytes (Figure 4, Table 1) contrasted the compromised expression of the chemokines *IL-8 *(neutrophil chemoattractant), *Gro-α/CXCL1 *(neutrophil chemoattractant), *MCP-2/CCL8 *(monocyte, lymphocyte, basophil, eosinophil chemoattractant) and *MIP-3α/CCL20 *(lymphocyte chemoattractant) (Table 1). The expression of *MDC/CCL22 *was found to be restricted to CD14^+ ^PBMC (monocytes) and expression in IRAK4-deficient monocytes was equivalent to or greater than expression in control monocytes (Figure 4B). ELISA was employed to demonstrate that LPS (24 hr) induced the secretion of 3-fold more MDC (3.3 ± 0.6) by the patient's PBMC compared to PBMC from adult or age-matched children (Figure 4C). Other CC family members including *MPIF/CCL23 *and *CCL28 *(chemotactic for monocytes, CD4^+ ^and CD8^+ ^T cells) and the chemokine receptor *CXCR3 *were also expressed at normal levels in IRAK4-deficient cells (ArrayExpress E-FPMI-7). Given that monocytes, dendritic cells, NK cells, and activated T cells are all chemoattracted by MDC/CCL22, the heightened expression of this chemokine alone has the potential to recruit essential immune cells and thus substantially compensate for the collective absence of chemokines Gro-α/CXCL1, MCP-2/CCL8, MIP-3α/CCL20 and IL-8.

### Gene expression in IRAK4-deficient monocytes was subject to transcriptional and translational regulation

Induced genes in LPS-stimulated, IRAK4-deficient monocytes were likely the result of signal transduction via the MyD88/IRAK4-independent, TRIF/TRAM-dependent arm of the TLR4 pathway. Clearly however, LPS-induced activation of the MyD88/IRAK4-independent pathway did not completely compensate for a defect in the MyD88-dependent pathway, neither in the number of expressed genes nor magnitude of their expression.

**Figure 5:**
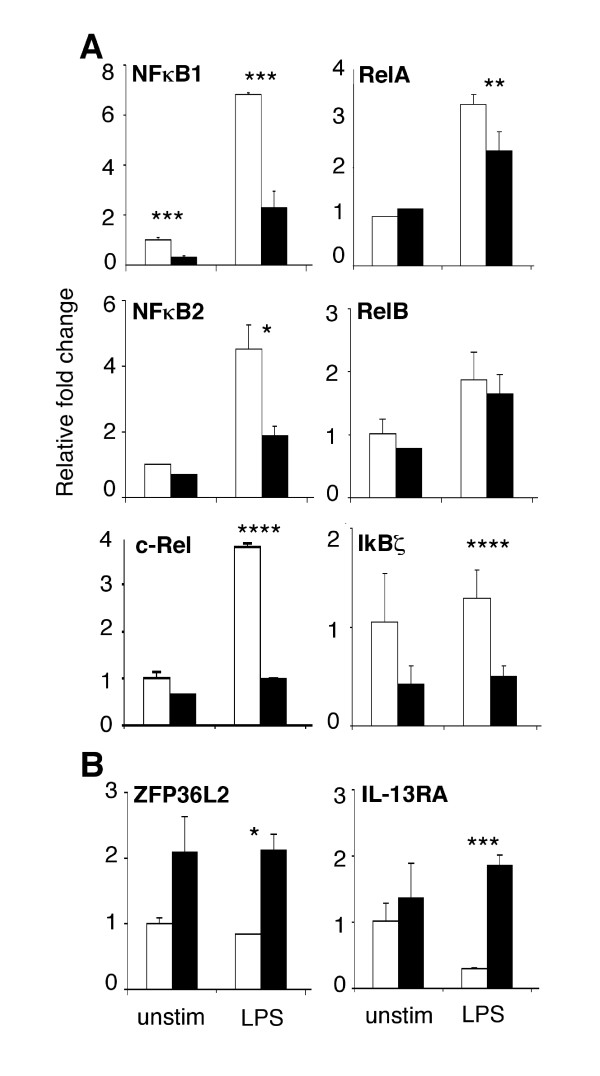
**Differential regulation of elements that regulate gene transcription and translation in IRAK4-deficient monocytes**. The relative expression (Y-axis) of (**A**) NFκB subunits NFκB1 (p105/50), NFκB2 (p100/52), RelA (p65), RelB & c-Rel, NFκB regulator IκBζ, and (**B**) translational suppressors IL-13RA & ZFP36L2 in IRAK4-deficient monocytes (black bars) and controls (white bars) was measured by real-time PCR following 4 hr incubation in the absence or presence of LPS (X-axis). Results show gene expression relative to unstimulated control monocytes and are representative of 3 independent experiments. Differences in expression were determined to be statistically significant by Student's two tailed T-test with a value of p < 0.15 (*), p < 0.1 (**), p < 0.05 (***) or p < 0.01 (****).

Kinetic models have estimated that MyD88/IRAK4-independent activation of NFκB via TRIF/TRAM lags the MyD88-dependent pathway by approximately 30 min [38]. We and others previously demonstrated that cells from the IRAK4-deficient patient [13,14] and MyD88-deficient mice [39] do indeed activate NFκB shortly after exposure to LPS. The depletion of cytoplasmic pools of NFκB as a consequence of nuclear translocation stimulates new transcription of NFκB subunits in order to replenish cytoplasmic supplies. This phenomenon was observed here (Figure 5A, Table 1) for LPS-activated control monocytes that induced the compensatory expression of all five NFκB subunits: *NFκB1/p50*, *NFκB2/p52*, *RelA/p65*, *RelB *and *c-Rel*. The compensatory expression of all subunits except *RelB *was however diminished in IRAK4-deficient cells. The expression of *NFκB1/p50 *and *c-Rel *was significantly (p < 0.05) more impaired after LPS activation than two other subunits (*NFκB2/p52 *and *RelA/p65*). Likewise, *IκBζ*, a LPS-inducible regulator of NFκB that is required for IL-6 transcription [40] was expressed at sub par levels in IRAK4-deficient monocytes (Figure 5A, Table 1), which correlated with the compromised expression of IL-6 (Figure 3, Table 1). These data offer evidence that an initial wave of normal NFκB activation and gene transcription in IRAK4-deficient cells (via the TRIF/TRAM pathway) [13,14] would be prematurely truncated due to a substantial deficit in the ability of NFκB to induce its own compensatory transcription in response to LPS.

The expression of cytokines, chemokines and transcription factors is also heavily dependent on the stability of transcribed mRNA. AU-rich elements (ARE) are found near the 3' untranslated region (UTR) of these mRNAs and target the message for degradation. The zinc finger protein ZFP36L2, a relative of the prototypic Zinc-finger protein tristetraprolin/TTP, targets the ARE region of TNF-α mRNA, destabilizes the sequence and prevents secretion of TNF-α [41]. The expression of *ZFP36L2 *was elevated greater than 2-fold in IRAK4-deficient monocytes compared to control monocytes (Figure 5B, Table 1), a result consistent with the compromised expression of TNF-α mRNA and protein (Figure 3). Another negative regulator of TNF-α, IL-13RA1, was upregulated more than 6-fold in LPS-stimulated IRAK4-deficient monocytes (Figure 5B, Table 1). IL-13RA1 mediates translational repression of TNF-α mRNA in LPS-stimulated monocytes and is required for IL-13-mediated protection of mice from lethal endotoxemia [42]. Other mRNA (de)stabilization agents may be direct or indirect substrates of IRAK4, for example, the MAPKs that also act on ARE-binding proteins such as TTP were severely impaired in IRAK4-KD BMDM [37].

These results suggest that the expression of NFκB-regulated genes in IRAK4-deficient cells results from the combined contribution of transcriptional regulation of the gene itself as well as that of other genes that encode elements that influence transcriptional longevity (e.g., NFκB) and mRNA stability (e.g., *ZFP36L2, IL-13RA1*). These data offer an explanation for the variable expression, rather than abolishment, of mRNA/protein of various cytokines and chemokines in IRAK4-deficient cells. Furthermore, these data demonstrated that the observed gene expression is regulated and the reciprocal relationships between certain genes (e.g., over-expression of ZFP36L2 & IL13RA1 with repression of TNF-α) is preserved, albeit in an unbalanced state compared to IRAK4-competent cells. These data indicate that an initial, but perhaps not subsequent waves of inflammatory mediators are produced in the absence of IRAK4, a result that is in line with a previous suggestion that IRAK4 may only become important in the face of a sustained bacterial challenge [37].

## Discussion

Individuals with defects in key immunity genes, while rare, represent valuable models for understanding the immune system and disease pathogenesis in humans. Sub-optimal expression of particular TLR-responsive genes has become an accepted hallmark of a primary immunodeficiency and a predisposition to infection. In contrast to previous studies that evaluated a selected subset of immunity genes, this study employed microarray technology to evaluate the impact of an IRAK4-deficiency on 21 K human genes. The absence of IRAK4 compromised the appropriate expression of 567 LPS-responsive genes in human monocytes, including a broad array of genes (pro-inflammatory interleukins, CXC and CC chemokines, and NFκB subunits) implicated in inflammation and immunity. In accordance with previous analyses on a much smaller subset of genes and proteins, the global disruption of gene expression in IRAK4-deficient cells conclusively demonstrated that IRAK4 plays a pivotal role in transmission of signals along the TLR4 pathway that culminate in robust pro-inflammatory gene transcription. Based on the current belief that robust TLR4-responsiveness is a requirement for immunity, these data would also have led us to predict that IRAK4-deficient children would be highly susceptible to Gram negative infections.

The clinical data, however, indicate that invasive infection by Gram negative bacteria are at most a rare occurrence in IRAK4-deficient children despite severely compromised responses of IRAK4-deficient cells to LPS. To confirm this phenotype in a controlled situation, we utilized IRAK4-deficient mice to quickly ascertain their ability to survive infection by a Gram negative bacterium *in vivo*. These mice, like IRAK4-deficient children, are susceptible to infections by Gram positive bacteria and fail to produce inflammatory cytokines in response to LPS [19-24]. We now demonstrate that IRAK4 knockout mice and wildtype mice have a similar resistance to challenge with Gram negative bacteria, thereby providing strong evidence that effective immunity is maintained and may account for the relatively few cases of Gram negative infections in mice and children with an IRAK4-deficiency.

These data are consistent with the hypothesis that a robust transcriptional response to LPS is not essential as long as key aspects of the immune response are maintained; such a hypothesis must be qualified given that the complete absence of such responses in MyD88 knockout mice does influence susceptibility to Gram negative infections. Such subtle changes in the elements of the immune system, including for e.g., polymorphisms in certain genes are becoming increasingly recognized as sufficient to alter immunity [43]. For example only 250 genes are included within the major locus of variability between *S. pneumoniae*-sensitive and -resistant strains of mice [44]. During the course of this study, we also evaluated the transcriptional responsiveness of IRAK4-deficient cells to peptidoglycan (PGN). This component of the cell wall of Gram positive bacteria stimulates several innate immunity receptors, including TLR2. Despite a predisposition of IRAK4-deficient children to Gram positive infections, we observed relatively normal transcriptional responses of IRAK4-deficient monocytes to PGN (data included in ArrayExpress E-FPMI-7). These data also suggested to us that immunity is quite complex and cannot necessarily be inferred from even a global assessment of agonist-induced transcriptional responses.

If one accepts at least a portion of the prevailing dogma and assumes that essential elements of Gram negative immunity are contained within the transcriptional response to LPS, it is possible to speculate, based on the data provided here, that these key elements either (1) include classical pro-inflammatory cytokines and chemokines that adequately perform immunological function(s) at reduced concentrations (e.g., TNF-α, IL-6) or (2) are contained within the subset of genes that were expressed to similar levels in IRAK4-deficient and control monocytes (e.g., MDC/CCL22, DEFB1). Consistent with these concepts, it was recently demonstrated that innate defence regulator peptides, which suppress pro-inflammatory cytokines but substantially maintain chemokine responses, are able to protect against *Salmonella *infections [30]. The functional redundancy of chemokines is an example of how the expression of just a few genes might be sufficient to support critical immunological functions such as cellular recruitment, despite the severe impairment of the expression of other family members. As demonstrated here, the expression of IL-8/CXCL8, *Gro-α/CXCL1*, MCP-2/CCL8 and MIP-3α/CCL20 was compromised in LPS-stimulated IRAK4-deficient monocytes (Figure 3, Table 1), suggesting a diminished recruitment of neutrophils, monocytes, lymphocytes, basophils and eosinophils to sites of infection. However, the chemokines MDC/CCL22, MPIF/CCL23, CCL28 and DEFB1 were robustly expressed in the absence of IRAK4 (Figure 5 and ArrayExpress), implying that monocytes, dendritic cells, natural killer cells, memory T cells, and activated CD4^+ ^and CD8^+ ^T cells, particularly CD4^+ ^T-helper-2 (Th2) cells could still be mobilized in response to LPS. The enhanced recruitment of Th2 cells that express IL-4 and IL-13 would be consistent with the elevated expression of IL-13RA1 (Figure 5), as well as reports of relatively normal antibody responses to vaccination in some children with IRAK4-deficiency [15,16]. Similar to our findings in human IRAK4-deficient monocytes, a number of chemokines were expressed at similar levels in murine BMDM from wildtype and IRAK4-kinase-defective knock-in mice, such as CXCL2, CXCL10, CXCL11, CCL2 and CCL4 [Clusters I and III in [37]]. We propose that the observed differential expression of chemokines is sufficient to appropriately change the cellular milieu at the site of infection and favourably impact on the outcome to infection [45]. Without supporting *in vivo *data however, these results are at best speculative. It should however be noted that infections in patients with IRAK4-deficiency are pyogenic (pus-forming) and can lead to mild fever and inflammation at late stages of infection [12]. This *in vivo *evidence implies that certain inflammatory mediators must have been produced and that cells are actively recruited to the site of infection in IRAK4-deficient individuals. Furthermore, TNFα and IL-6 were detected, albeit at low levels in the serum of IRAK4-KD mice following administration of LPS [37].

Other potential candidate elements that might mediate defence include the >60 upregulated genes and 20 down regulated genes listed in Table S1, Additional file 1 (genes with similar or exaggerated expression in IRAK4-deficient cells compared to controls). Some of these genes in the ADAM, ICAM, integrin and NFκB families were also regulated in a similar fashion in IRAK4-KD and control BMDM stimulated with LPS [37]. Although GO analysis did not predict a disturbance in any major biological process in resting (unstimulated) IRAK4-deficient monocytes, we identified more than 50 LPS-responsive genes that were expressed in resting IRAK4-deficient cells not exposed to LPS (Table S2, Additional file 2). Notable genes include cytokines & chemokines (*IL1F9, MIP-2α/CXCL2*), transcription factors & transcriptional regulators (*c-REL, NFKB1A, IBRDC2, UBE2N, USP9Y, BACH1*), cell adhesion molecules (*CD44*), signalling molecules (*MAPK6, PRKAG2, CALM3*) and other pro-inflammatory mediators (*ALOX5, HIF3α, HLA-DMA, LILRA3*). It is tempting speculate that these differentially expressed genes in unstimulated, IRAK4-deficient cells may also contribute to successful host defences.

LPS-induced gene expression in IRAK4-deficient cells is subject to regulation by both transcriptional and translational mechanisms and is the product of signal transduction via either (1) the MyD88-independent, TRIF/TRAM pathway or (2) MyD88-dependent, IRAK4-independent pathways. Björkbacka and colleagues have demonstrated that only 20% of more than 1000 LPS-responsive genes in macrophages are in fact dependent on MyD88 [46]. We however favour the hypothesis that a subset of MyD88-dependent, IRAK4-independent genes are imperative for immunity since MyD88-deficient mice are susceptible to a broader range of pathogens, including Gram negative bacteria, than are caused by IRAK4-deficiency in mice and humans. MyD88-dependent pathways that transduce signals independent of IRAK4 utilize signalling molecules such as PI3K, Btk, Tlp-2, and NIK and activate MAPKs and NFκB. Regardless, it can be concluded that pathways other than the classic MyD88-dependent pathway (via sequential activation of IRAK4, IRAK1, TRAF6, IKK and NFκB) have essential and under-appreciated roles in defence against Gram negative bacteria.

## Conclusions

In this study, we demonstrated that the expression of the vast majority of LPS-induced inflammatory and immunity genes were compromised (compared to the expression levels in control cells) in monocytes from a child with IRAK4 deficiency. We conclude that while IRAK4 is imperative for a comprehensive transcriptional response to LPS, neither IRAK4, nor this classical, robust transcriptional response is required for effective host defences against Gram negative infection. Instead, the data implies that sufficient defence could lie within a small repertoire of LPS-responsive, IRAK4-independent genes. A subset of transcribed genes amidst a severely impaired response to LPS was also observed by Koziczak-Holbro *et al *in murine BMDM from IRAK4-kinase-defective knockin mice [37]. These observations are consistent with the concept that it may be possible to generate an effective immune response without a robust inflammatory response [47] and such properties may even make IRAK4 an attractive drug target for treating inflammation without compromising effective immune defences [48].

## Competing interests

The authors declare that they have no competing interests.

## Authors' contributions

KLB carried out the *in vitro *cell culture, RNA isolation, qPCR, ELISA and manuscript preparation. RF carried out the microarray analysis. WK carried out the animal studies. JLG performed bioinformatics analyses. PH added valuable insight into data analysis, manuscript content and presentation. DJD, ST and BBF provided advice on the conception and design of the clinical and animal experiments. DPS and REWH were imperative to the conception, design and implementation of the experiments and preparation of the manuscript. All authors read and approved the final manuscript.

## Supplementary Material

Additional file 1 **Table S1**. Selection of LPS-responsive immunity genes that were expressed to similar or exaggerated (italics) levels in IRAK4-deficient monocytes compared to controls as assayed by microarray analysis Click here for file

Additional file 2 **Table S2**. Selection of LPS-responsive genes expressed in unstimulated IRAK4-deficient monocytes Click here for file
