# Supplementary material for: Robust TLR4-induced gene expression patterns are not an accurate indicator of human immunity
Source: J Transl Med. 2010 Jan 27;8:6. doi: 10.1186/1479-5876-8-6 (PMC2843650; doi:10.1186/1479-5876-8-6)
Supplement: Additional file 1 — Table S1. Selection of LPS-responsive immunity genes that were expressed to similar or exaggerated (italics) levels in IRAK4-deficient monocytes compared to controls as assayed by microarray analysis [file 1479-5876-8-6-S1.PDF]

**Table S1.** Selection of LPS-responsive immunity genes that were expressed to similar or exaggerated (italics) levels in IRAK4-deficient monocytes compared to controls as assayed by microarray analysis.

| Gene Name | Description |
|---|---|
| **LPS-induced genes** | |
| ABL2 | Tyrosine-protein kinase ABL2 |
| ADAMTS6 | ADAMTS-6 precursor; A disintegrin and metalloproteinase |
| ADPRHL1 | ADP-ribosylhydrolase like 1 isoform 1 |
| *ALOX5* | *Arachidonate 5-lipoxygenase* |
| ANAPC5 | Anaphase-promoting complex subunit 5 |
| *CAMSAP1L1* | *Calmodulin regulated spectrin-associated protein 1-like 1* |
| CAPZB | F-actin capping protein subunit beta |
| *CCL23* | *Small inducible cytokine A23 precursor (CCL23/MIP-3/MPIF-1)* |
| CCNB1IP1 | E3 ubiquitin-protein ligase CCNB1IP1 |
| *CCS* | *Copper chaperone for superoxide dismutase* |
| CDC2L6 | Cell division cycle 2-like protein kinase 6 |
| CLK3 | Dual specificity protein kinase CLK3 |
| COL9A2 | Collagen alpha-2(IX) chain precursor. |
| *CORO1B* | *Coronin-1B (Coronin-2)* |
| CYB5B | Cytochrome b5 type B precursor |
| DDX3X | ATP-dependent RNA helicase DDX3X |
| DNAH5 | Ciliary dynein heavy chain 5 |
| DSTN | Destrin (Actin-depolymerizing factor) |
| EPHB2 | Ephrin type-B receptor 2 precursor; Tyrosine-protein kinase receptor EPH-3) |
| FBN1 | Fibrillin-1 precursor |
| *FEZ2* | *Fasciculation and elongation protein zeta 2 (Zygin-2)* |
| GALNT9 | Polypeptide N-acetylgalactosaminyltransferase 9 |
| HBG2 | Hemoglobin subunit gamma-2 |
| HIPK2 | Homeodomain-interacting protein kinase 2 |
| HNRPLL | Heterogeneous nuclear ribonucleoprotein L-like (Stromal RNA-regulating factor) |
| HNF4A | Hepatocyte nuclear factor 4-alpha (HNF-4-alpha); Transcription factor HNF-4 |
| ICAM3 | Intercellular adhesion molecule 3 |
| *IDH3G* | *Isocitrate dehydrogenase [NAD] subunit gamma; Isocitric dehydrogenase* |
| IER3 | Radiation-inducible immediate-early gene IEX-1 |
| IGHG3 | Immunoglobulin heavy chain C gene segment |
| *KCNK6* | *Potassium channel subfamily K member 6* |
| *KIAA0859* | *CGI-01 protein isoform 2* |
| KPNA4 | Importin alpha-4 subunit (Karyopherin alpha-4 subunit) |
| KPNB1 | Karyopherin beta-1 subunit, Nuclear factor P97/Importin 90 |
| Magmas | Mitochondria-associated GM-CSF-signaling molecule |
| MAK10 | corneal wound healing-related protein |
| MAN2B1 | Lysosomal alpha-mannosidase precursor |
| *MKKS* | *McKusick-Kaufman/Bardet-Biedl syndromes putative chaperonin* |
| MPP4 | MAGUK p55 subfamily member 4 (Discs large homolog 6) |
| NME2 | Nucleoside diphosphate kinase B (C-myc purine-binding transcription factor PUF) |
| NPAS2 | Neuronal PAS domain-containing protein 2 |
| *NRXN1* | *Neurexin-1-beta precursor (Neurexin I-beta)* |
| NUBP1 | Nucleotide-binding protein 1 (NBP 1) |
| PDCD11 | RRP5 protein homolog (Programmed cell death protein 11) |
| PFAAP5 | phosphonoformate immuno-associated protein 5 |

| | |
|---|---|
| PKP4 | Plakophilin-4 (p0071) |
| PLEKHB2 | Pleckstrin homology domain-containing family B member 2 (Evectin-2) |
| PRLR | Prolactin receptor precursor (PRL-R) |
| *RCL1* | *RNA 3'-terminal phosphate cyclase-like protein* |
| *SBNO1* | *sno, strawberry notch homolog 1* |
| SELT | Selenoprotein T precursor |
| SHOX2 | Short stature homeobox protein 2 |
| SIDT1 | SID1 transmembrane family member 1 precursor. |
| SLC39A8 | solute carrier family 39 (zinc transporter) |
| SMEK1 | SMEK homolog 1. |
| SMPDL3A | Acid sphingomyelinase-like phosphodiesterase 3a |
| *SMURF2* | *Smad ubiquitination regulatory factor 2* |
| SSBP3 | Single-stranded DNA-binding protein 3 |
| STAMBP | STAM-binding protein |
| SUV420H1 | suppressor of variegation 4-20 homolog 1 |
| *TDRD7* | Tudor domain-containing protein 7 |
| THRAP2 | Thyroid hormone receptor-associated protein 2 |
| *TMEM107* | *Transmembrane protein 107* |
| TOR1AIP2 | Torsin-1A-interacting protein 2 |
| TRAC | T cell receptor alpha constant |
| TRAPPC4 | Trafficking protein particle complex subunit 4 (Synbindin) |
| TREX1 | ATR-interacting protein (ATM and Rad3-related-interacting protein) |
| TSPAN11 | tetraspanin 11 |
| ZGPAT | Zinc finger CCCH-type with G patch domain protein |
| ZHX2 | Zinc finger and homeodomain protein 2 |
| ZNF185 | Zinc finger protein 185 |
| ZNF197 | Zinc finger protein 197 (ZnF20) |
| **LPS-repressed genes** | |
| ARL14 | ADP-ribosylation factor 7 |
| ARID3A | AT-rich interactive domain-containing protein 3A (B-cell regulator of IgH transcription) |
| *BEST3* | *Bestrophin-3 (Vitelliform macular dystrophy 2-like protein 3)* |
| *CALM3* | *Calmodulin (CaM)* |
| CBX5 | Chromobox protein homolog 5 |
| CCDC28A | Coiled-coil domain-containing protein |
| CTAGE5 | Cutaneous T-cell lymphoma-associated antigen 5 (cTAGE-5 protein) |
| FGD3 | FYVE, RhoGEF and PH domain-containing protein |
| ITGB6 | Integrin beta-6 precursor |
| PPT1 | Palmitoyl-protein thioesterase 1 precursor |
| PRMT3 | Protein arginine N-methyltransferase 3 |
| PTGER2 | Prostaglandin E2 receptor, EP2 subtype |
| RAB28 | Ras-related protein Rab-28 (Rab-26) |
| RIPK3 | Receptor-interacting serine/threonine-protein kinase 3 |
| SKP1A | S-phase kinase-associated protein 1A |
| SVIL | Supervillin (Archvillin) |
| TCF7L2 | Transcription factor 7-like 2 (HMG box transcription factor 4) |
| USP9Y | Probable ubiquitin carboxyl-terminal hydrolase FAF-Y |
| *VDAC1* | *Voltage-dependent anion-selective channel protein 1* |
| ZNF442 | Zinc finger protein 442 |
