# Supplementary material for: Robust TLR4-induced gene expression patterns are not an accurate indicator of human immunity
Source: J Transl Med. 2010 Jan 27;8:6. doi: 10.1186/1479-5876-8-6 (PMC2843650; doi:10.1186/1479-5876-8-6)
Supplement: Additional file 2 — Table S2. Selection of LPS-responsive genes expressed in unstimulated IRAK4-deficient monocytes [file 1479-5876-8-6-S2.PDF]

Table S2. Selection of LPS-responsive genes expressed in unstimulated IRAK4-deficient monocytes

Gene Name	Description
ADAMTS20	ADAMTS-20; A disintegrin and metalloproteinase
ACTR2	Actin-like protein 2
ADPRHL1	ADP-ribosylhydrolase like 1 isoform 1
ALOX5	Arachidonate 5-lipoxygenase, important in inflammation and hypersensitivity.
AQP9	Aquaporin-9; Small solute channel 1
ARTN	Artemin precursor
ATG16L2	ATG16 autophagy related 16-like 2
ATXN7L3	ATXN7L3 protein
BACH1	Transcription regulator protein BACH1 (BTB and CNC homolog 1)
BAZ2A	Bromodomain adjacent to Zn-finger protein 2A; Transcription termination factor
BEST3	Bestrophin-3
BMP15	Bone morphogenetic protein 15 precursor (BMP-15)
CACNA1H	Voltage-dependent T-type calcium channel subunit alpha-1H
CALM3	Calmodulin
CD44	CD44 antigen; cell adhesion molecule
CXCL2	Macrophage inflammatory protein 2-alpha; chemokine
DDX3X	ATP-dependent RNA helicase
DEPDC5	DEP domain-containing protein 5
DNAH5	Ciliary dynein heavy chain 5
DNAJB2	DnaJ homolog subfamily B member 2; Heat shock 40 kDa protein 3
DPH4	DPH4 homolog; CSL-type zinc finger-containing protein 3
FAM126A	Protein FAM126A; Down-regulated by CTNNB1 protein A
FTCD	Formimidoyltransferase-cyclodeaminase
HBG2	Hemoglobin subunit gamma-2
HIF3A	hypoxia-inducible factor-3 alpha isoform c
HLA-DMA	HLA class II histocompatibility antigen, DM alpha chain precursor
HNRPLL	Heterogeneous nuclear ribonucleoprotein L-like; Stromal RNA-regulating factor
IBRDC2	E3 ubiquitin-protein ligase IBRDC2 ; p53-inducible RING finger protein
IER3	Radiation-inducible immediate-early gene IEX-1; Immediate early protein GLY96
IL1F9	Interleukin-1 family member 9
KPNA4	Importin alpha-4 subunit
LILRA3	Leukocyte immunoglobulin-like receptor subfamily A member 3
MAK10	corneal wound healing-related protein
MAPK6	Mitogen-activated protein kinase 6
MEF2C	Myocyte-specific enhancer factor 2C
MFSD2	Major facilitator superfamily domain-containing protein 2
MYL4	Myosin light polypeptide 4
NFKBIA	NF-kappa-B inhibitor alpha
NOV	Protein NOV homolog
NUP155	Nuclear pore complex protein
PRKAG2	5'-AMP-activated protein kinase subunit gamma-2
RCL1	RNA 3'-terminal phosphate cyclase-like protein
REL	C-Rel proto-oncogene protein; NFkB subunit
RPAIN	RPA-interacting protein
S100A12	Protein S100-A12; S100 calcium-binding protein A12, ligand for RAGE
SIRT1	NAD-dependent deacetylase sirtuin-1; stress-response and chromatin-silencing factor

SLAMF8	SLAM family member 8; B-lymphocyte activator, macrophage expressed
SLC2A3	Solute carrier family 2, facilitated glucose transporter member 3
SORBS2	Sorbin and SH3 domain containing 2 isoform 2
SPPL2A	Signal peptide peptidase-like 2A
SV2B	Synaptic vesicle glycoprotein 2B
TMEM134	transmembrane protein 134
UBE2N	Ubiquitin-conjugating enzyme E2 N; required to induce and immune response
USP9Y	Probable ubiquitin carboxyl-terminal hydrolase FAF-Y
ZNF442	Zinc finger protein 442
